# Human‐Centered Innovation: Precision Nutrition and the Future of Food

**DOI:** 10.1002/advs.202523337

**Published:** 2026-07-07

**Authors:** Shanshan Zhang, Yonggan Sun, Jiaqi Jiang, Shaoping Nie

**Affiliations:** ^1^ State Key Laboratory of Food Science and Resources, China‐Canada Joint Lab of Food Science and Technology Key Laboratory of Bioactive Polysaccharides of Jiangxi Province Nanchang University Nanchang China

**Keywords:** artificial intelligence, food ingredients, food innovation, human‐centered design, omics, personalized nutrition, precision nutrition

## Abstract

Nutritional science is moving beyond one‐size‐fits‐all recommendations toward more precise and personalized approaches, yet its implementation pathway remains unclear. This paper proposes a human‐centered precision nutrition framework that highlights the central roles of artificial intelligence (AI), food innovation, and human agency. By integrating multidimensional data, including clinical phenotypes, multi‐omics, lifestyle, and personal context, AI can assess health status to identify “what needs to be regulated” and predict dietary responses based on baseline characteristics to determine “what should be supplemented”, thereby supporting more precise dietary predictions and recommendations. However, because people are dynamic social beings, precision nutrition must also account for real‐world constraints, including habits, time, finances, emotions, and daily routines. Therefore, dietary recommendations should be translated into diverse options that support personal autonomy in deciding “how to supplement”. As important carriers of intervention, food materials may range from whole foods and natural ingredients to functional extracts, probiotics, and rationally designed functional components. Technological innovation can further translate these materials into diverse delivery formats and service models that better fit daily life. Looking ahead, precision nutrition should be guided by human‐centered design, translating scientific and technological advances into practical, accessible, and sustainable dietary strategies that become part of everyday life.

## Graphical abstract

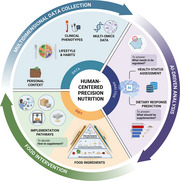

## 1 Introduction

Diet and nutrition are fundamental to human health and are closely linked to the development and progression of chronic diseases [1]. Conventional public nutrition guidelines and one‐size‐fits‐all dietary recommendations are inherently based on population averages, and therefore exhibit significant limitations in their applicability at the individual level [2]. Given the substantial variation in genetic background, metabolic phenotype, gut microbiota composition, and lifestyle, physiological responses to the same food can vary markedly [3]. Against this background, precision nutrition has emerged, aiming to move beyond the limitations of population averages and provide tailored nutritional interventions based on personal characteristics. With the rapid development of omics technologies, including genomics, microbiomics, and metabolomics, as well as the adoption of digital tools such as wearable devices and continuous glucose monitoring, precision nutrition is moving from concept to practice. It is regarded as an important frontier for the prevention and management of chronic metabolic diseases and the promotion of public health [4, 5].

In this rapidly evolving field, the terms “precision nutrition” and “personalized nutrition” are often used interchangeably. Based on the existing literature, personalized nutrition typically provides relatively broad dietary guidance based on information such as phenotypic characteristics, nutrigenomics, behavioral habits, and lifestyle factors [6, 7, 8]. Precision nutrition, by contrast, goes a step further by integrating data, including genetics, dietary habits and eating patterns, circadian rhythms, health status, socioeconomic and psychosocial characteristics, food environments, physical activity, and the microbiome [4]. It places greater emphasis on molecular‐level information and the interrelationships among these factors, while also focusing more strongly on dynamic and holistic changes [6, 7]. However, effectively linking precise prediction with daily life settings remains a key challenge in translating precision nutrition from theory into practice. This challenge highlights the need for a human‐centered approach to precision nutrition. As conceptualized in this article, human‐centered precision nutrition refers to a food‐based approach that uses artificial intelligence (AI)‐driven analysis to translate precise prediction into feasible, flexible, and sustainable dietary options. It integrates multidimensional data to support precise assessment of health status and prediction of nutritional needs, while ensuring that dietary recommendation design remains grounded in personal preferences and real‐world constraints, such as cultural practices, economic conditions, and lifestyle. By providing diverse food‐based intervention strategies and enabling individuals to actively participate in choosing dietary strategies through shared decision‐making, this approach seeks to balance scientific precision with humanistic adaptability and enhance the feasibility and long‐term sustainability of nutritional interventions.

Building on this, the article discusses the integration of multiple types of data and how AI can be used to generate more precise dietary recommendations. It also examines how food, as an important vehicle for intervention, can provide diverse solutions for precision nutrition. In addition, it analyzes the challenges in implementing human‐centered precision nutrition and outlines future research directions. Through these discussions, the article aims to provide a theoretical basis and practical guidance for developing a precision nutrition practice framework that respects individual differences and remains feasible in real‐world settings.

## 2 Understanding the Individual: Data and Intelligence in Precision Nutrition

Precision nutrition begins with a comprehensive understanding of human variability. Achieving precision dietary intervention requires accurate assessment of a person's health status, prediction of how that person responds to different foods, and consideration of lifestyle and daily life factors. Together, these objectives define the data foundation of a human‐centered precision nutrition framework.

### 2.1 Multidimensional Data as the Foundation of Individual Characterization

Clinical phenotypes and physiological feedback provide the most accessible evidence of health status. Routine measurements including blood glucose, blood lipids, blood pressure, and liver and kidney function markers establish a baseline metabolic profile and provide a foundation for evaluating nutritional needs and monitoring intervention outcomes. Traditional health examinations, however, capture only periodic snapshots rather than continuous physiological dynamics. The adoption of wearable technologies has begun to change this, as continuous glucose monitoring, for example, records the full trajectory of the postprandial glycemic responses to different foods, offering a more accurate reflection of metabolic capacity than a single measurement [9].

Beyond clinical indicators, multi‐omics data at the molecular level further illuminate the biological basis of interindividual variability. Genomics reveals specific genetic variants associated with disease susceptibility and specific nutritional requirements. From a nutrigenetic perspective, for example, the AMY1 gene is associated with carbohydrate metabolism capacity, and its copy number is negatively correlated with obesity risk [10], and variants in the MTHFR gene affect folate metabolism [11]. Databases such as dbSNP, dbGaP, and GWAS Central provide critical support for discovering and validating such gene‐phenotype and gene‐nutrient associations [12]. Epigenomics reveals how long‐term lifestyle factors alter gene expression through chemical modifications. Unhealthy diets and chronic stress have been associated with methylation changes at loci such as ABCG1 and SEL1L, potentially contributing to inflammation, insulin resistance, and lipid metabolic dysregulation [13]. Transcriptomics and proteomics record cellular‐level responses to nutrient intake, reflecting changes in key metabolic enzymes and signaling proteins and enabling the identification of disease risk factors [14, 15]. Metabolomics provides a dynamic biochemical readout for precision nutrition by capturing both dietary intake and endogenous metabolic status. Food‐intake biomarkers improve the objectivity of dietary assessment [16], and changes in endogenous metabolites can indicate early metabolic risk, as shown by the association of elevated taurocholic acid and reduced glycine with type 2 diabetes risk [17]. The gut microbiome, increasingly regarded as “the second genome” of the human body, interacts closely with host metabolism, immunity, and even the nervous system. Its value lies in both health assessment and response prediction. Dysbiosis often precedes clinical disease onset, providing early warning signals of intestinal barrier dysfunction or systemic inflammation [18]. More importantly, the microbiome represents a core explanatory variable underlying metabolic heterogeneity. Because microbial communities differ in their capacity to metabolize dietary fiber, polyphenols, and other bioactive compounds, the microbiome profiles can determine responses to dietary interventions [19, 20]. Even under matched macronutrient intake, differences in gut microbiota can lead to markedly distinct postprandial glucose responses and clinical parameter fluctuations [3, 21]. With the continued development and integration of omics technologies, our understanding of health and nutrition is being reshaped, marking a transition toward molecularly informed personalized interventions.

Lifestyle and personal context constitute an equally indispensable dimension. Eating rhythms, sleep quality, physical activity, and psychological stress are not merely background factors; they actively modulate metabolic responses to food through their effects on circadian regulation, neuroendocrine status, and gut microbial composition [22, 23]. Geographic location and seasonal food availability shape actual nutrient intake and microbiome composition [24]. Before formulating dietary recommendations, individual and contextual factors such as dietary preferences, cultural backgrounds, food availability, economic capacity, and occupational characteristics should be carefully considered, as they largely determine whether a recommendation is acceptable and sustainable over time.

### 2.2 AI‐Driven Pathways From Data to Dietary Guidance

The scale and diversity of data pose challenges for conventional statistical methods, while AI, particularly machine learning and deep learning approaches, provides an analytical framework for extracting meaningful signals from such complexity [25, 26, 27]. More specifically, AI serves as a tool for data integration, health risk assessment and dietary response prediction, and the translation of model outputs into personalized dietary recommendations. In 2015, Zeevi et al. developed a machine‐learning algorithm that integrated blood parameters, dietary habits, anthropometrics, physical activity, and gut microbiota to predict postprandial glycemic responses to meals, and validated its performance in an independent cohort [28]. Building on this work, subsequent studies combined machine‐learning prediction with mobile app‐based guidance to develop a personalized postprandial‐targeting (PPT) diet, which provided tailored dietary advice and improved postprandial glycemic responses and cardiometabolic markers in adults with prediabetes [29]. The PREDICT 1 study developed machine‐learning models incorporating phenotypic characteristics, genetic factors, meal composition, and gut microbiome data to predict postprandial triglyceride and glycemic responses [25]. More recently, the ZOE METHOD study used the ZOE 2022 algorithm, which incorporated food characteristics, personal postprandial glucose and triglyceride responses, microbiome profiles, and health history to generate personalized food scores and dietary recommendations through a mobile application; this app‐based personalized dietary program improved several cardiometabolic outcomes [30]. Together, these studies indicate that dietary interventions tailored to person‐specific characteristics can yield measurable physiological benefits in real‐world or near‐real‐world settings.

Beyond these studies, AI may also support a more upstream step in precision nutrition, which is health status assessment and intervention target identification. A comprehensive precision nutrition framework should first evaluate overall health status, identify latent disease risks and modifiable metabolic features, and then predict responses to specific dietary strategies. Machine learning and deep learning approaches have shown strong potential for identifying disease‐related risk features from multi‐omics data, including genomics, transcriptomics, proteomics, microbiomics, and metabolomics, thereby providing a methodological basis for health‐risk assessment in precision nutrition [31, 32, 33]. The PROGRESS prospective cohort demonstrated this potential by integrating continuous glucose monitoring, genomic data, gut microbiome profiles, electronic health records, wearable device metrics, dietary logs, and lifestyle information into a diabetes risk assessment model that outperformed traditional HbA1c‐based stratification [34]. The ZOE Microbiome Health Ranking links gut microbial taxa with diet‐ and health‐related markers, translating complex microbiome profiles into quantitative health‐related scores and identifying key species associated with metabolic risk, thereby providing a reference for prioritizing microbiome‐related intervention targets in precision nutrition [35]. However, findings from the TEDDY cohort serve as an important caution that microbiome data alone proved less predictive of type 1 diabetes than conventional clinical indicators [36]. This suggests that AI‐based health status assessment should not rely on any single omics layer, but should integrate multi‐omics risk signals with clinical information to identify actionable targets and guide subsequent dietary response prediction.

Dietary response prediction represents another important role of AI in precision nutrition. A growing body of evidence suggests that several factors, including the genome, microbiome, and metabolome, can influence the outcomes of dietary interventions, such as microbiome remodeling, metabolite changes, and improvements in clinical endpoints including glycemic responses [21, 37, 38]. Such biological complexity may lead to responder and non‐responder patterns, in which the same dietary intervention produces different effects across individuals. Therefore, precision nutrition requires both the identification of intervention targets and the prediction of how different people will respond to specific dietary strategies. Recent studies have demonstrated the potential of AI and computational models in this direction. The deep learning model McMLP predicted metabolite response to dietary intervention using baseline gut microbial composition and metabolite profiles [27]. In addition, microbial community‐scale metabolic modelling has been used to predict person‐specific short‐chain fatty acid production profiles and to evaluate the effects of different dietary, prebiotic, and probiotic inputs [39]. Together, for a given intervention target, AI may help predict how different foods or nutrition‐based strategies modulate that target, and may further support the design of personalized dietary interventions. For future applications, AI models should also estimate the expected efficacy, feasibility, and cost of different dietary strategies, while incorporating behavioral preferences and practical considerations. In addition, clinical, metabolic, and wearable‐derived feedback after dietary intervention could be used to dynamically update prediction models, forming a closed‐loop framework for precision nutrition. With the development of large language models, AI may further support knowledge integration, mechanistic interpretation, and natural‐language interaction, helping translate model‐based predictions into dietary strategies that users can understand and maintain.

**FIGURE 1:**
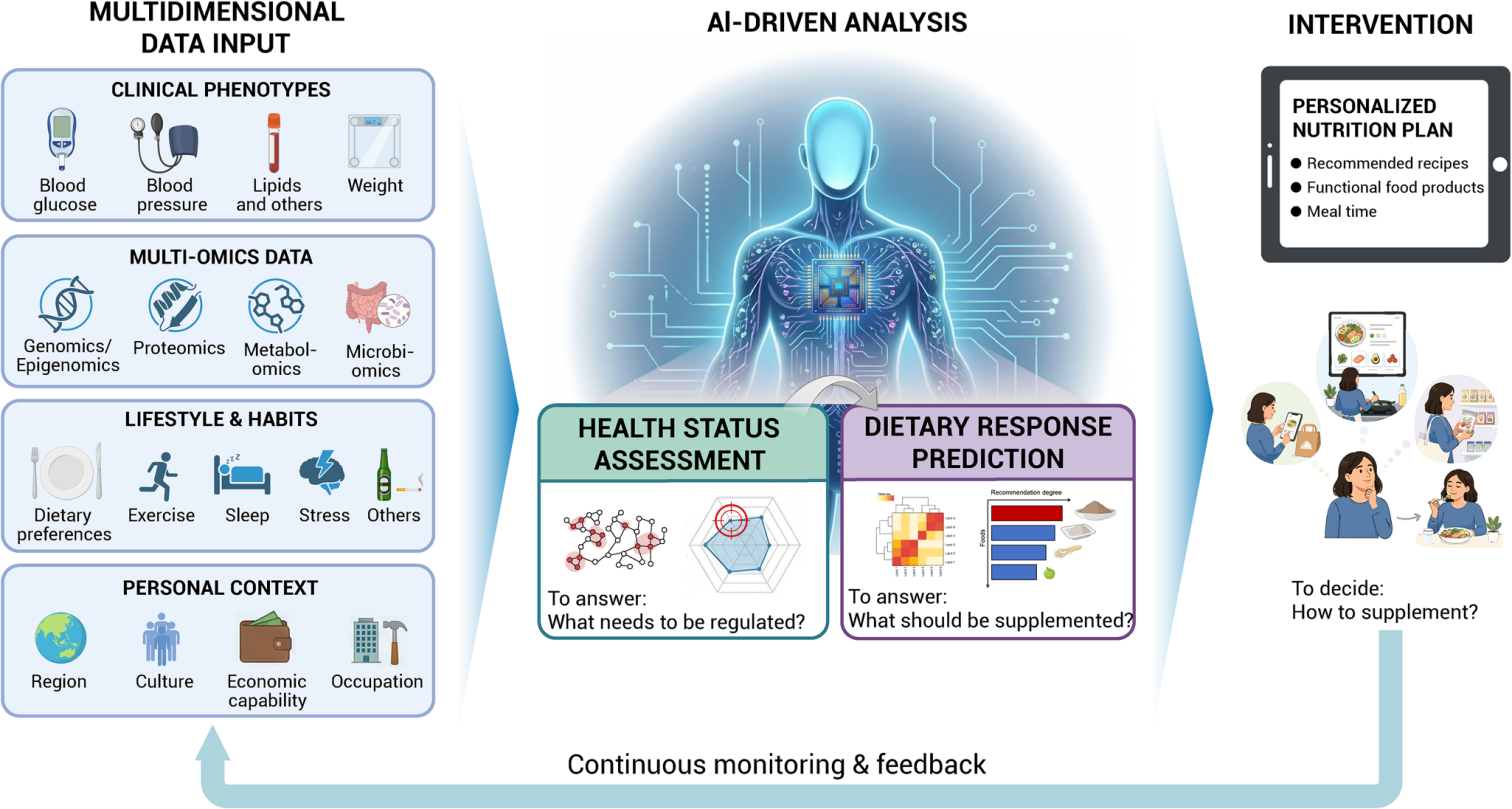
A human‐centered precision nutrition framework from data to table. Multidimensional data, including clinical, omics, lifestyle, and contextual information, are integrated to support AI‐based health status assessment, identification of intervention targets, and target‐specific dietary response prediction. By first identifying “what needs to be regulated” and then determining “what should be supplemented,” the framework generates personalized nutritional interventions and translates them into actionable options, allowing people to decide “how to supplement” in daily life. Continuous monitoring and feedback enable real‐time adaptation, iterative model refinement, and long‐term optimization of precision nutrition strategies.

Overall, broad data inputs and predictive models can improve the scientific precision of precision nutrition while helping translate model outputs into dietary strategies that reflect personal preferences and practical constraints (Figure 1). The final form of implementation, however, should remain guided by personal choice. In the future, the iterative process of data input, prediction, intervention feedback, and model updating may be implemented within a digital twin [40] framework, thereby reducing the cost of trial and error in practical settings. Nevertheless, several important limitations currently constrain the broader implementation of this paradigm. Heterogeneity in data formats and collection methods across platforms limits the transferability of models across populations. Large‐scale, high‐quality longitudinal multi‐omics datasets remain scarce, constraining both model training and validation. The interpretability of complex algorithms is a persistent challenge in clinical and personal application settings, where black‐box predictions are difficult to act on with confidence. Furthermore, prospective validation of model outputs is still insufficient, and the evidence base has not yet reached the robustness required to support widespread clinical translation.

## 3 Implementing Precision Nutrition: Diverse Food‐Based Solutions

**FIGURE 2:**
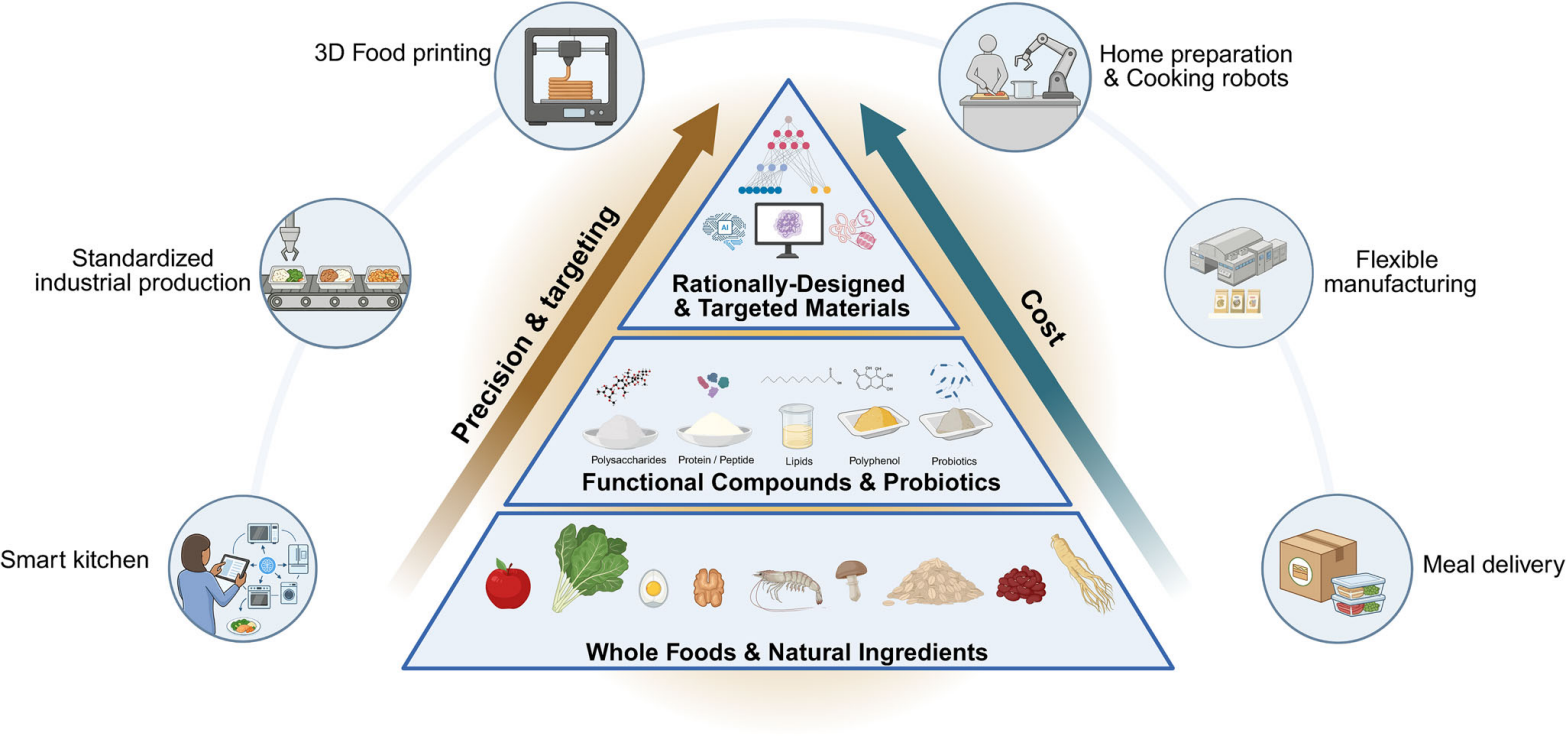
Food‐based solutions for precision nutrition. The ingredients and raw materials range from whole foods and natural ingredients to functional compounds, probiotics, and engineered ingredients, with increasing levels of precision and targeting accompanied by higher costs. The surrounding icons illustrate diverse implementation pathways, including industrial production, meal delivery services, and smart domestic systems, that enable precision nutrition to be translated into practical, personalized, and sustainable dietary solutions in everyday life.

The ultimate goal of precision nutrition is to translate scientific insights from health assessment and predictive modeling into actionable health behaviors in daily life. Food, as the main source of nutrients and bioactive compounds, is the practical medium for this translation. When clinically or biologically relevant targets are identified, such as glycemic or lipid phenotypes, microbial taxa, metabolites, or epigenetic alterations, specific food components can be used for targeted modulation. For example, elevated taurocholic acid and reduced glycine have been associated with type 2 diabetes risk, while fructooligosaccharides can regulate gut microbiota and bile acid profiles to activate the TGR5 pathway and improve lipid metabolism [17, 41]. Microbiome analyses have shown that patients with impaired glucose metabolism often have reduced butyrate‐producing bacteria, and dietary supplementation with resistant starch or complex fiber can modulate the gut microbiota, increase intestinal butyrate, and improve glucose metabolism via GPR41/43‐related pathways [42, 43, 44]. Dietary polysaccharides may also regulate epigenetic processes related to immunity, glucose and lipid metabolism, antioxidant defense, and antitumor activity [45]. These findings support the feasibility of targeted nutritional modulation based on the structure and function of food components. Considering variations in individual responses and practical constraints, a human‐centered precision nutrition framework should therefore provide diverse food‐based solutions (Figure 2).

### 3.1 Food Ingredients and Raw Materials

Food ingredients and raw materials form the foundation of dietary interventions. Among them, whole foods represent the most accessible and routinely consumed form of nutrition interventions, as they are widely available, culturally familiar, and easy to incorporate into long‐term eating habits. However, their health effects are also more difficult to predict with precision because whole foods are complex matrices composed of multiple nutrients and bioactive compounds. The interactions among these components, such as those between dietary fibers and polyphenols or between polyphenols and proteins, can influence nutrient bioavailability and physiological effects [46, 47]. Understanding such internal interactions is therefore essential for clarifying the health mechanisms of whole foods and improving the accuracy of prediction, although much work remains to be done. Prospective cohort studies are also important for defining the relationship between food intake and long‐term health outcomes. Studies have identified food types associated with specific health benefits, providing evidence base for constructing candidate functional food libraries in precision nutrition [48, 49]. At the same time, advances in analytical technologies allow more precise characterization of functional components in raw materials from different varieties and geographical origins [50]. Controlled‐environment agriculture, including vertical farming, may further improve the stability of nutrient profiles by optimizing growth conditions [51]. Within a precision nutrition framework, AI needs to integrate food composition, component interactions, and health‐outcome evidence to support more personalized and quantitative food recommendations. In this way, whole foods can serve as a practical and increasingly predictable foundation for precision nutrition.

When interventions based on whole foods lack sufficient potency or convenience, functional ingredients and probiotic interventions may provide more concentrated and controllable alternatives. Compared with whole foods, functional extracts often have more defined bioactive components, clearer dose ranges, and more tractable mechanisms of action. Modern extraction techniques enable efficient isolation of functional components from plants and organisms, including proteins and peptides, dietary polysaccharides, and polyphenols. Crucially, the integration of AI is accelerating this process, optimizing extraction protocols and facilitating the structural elucidation of complex molecules [52]. Many functional ingredients have shown potential for targeted modulation. Protein hydrolysates, such as whey‐derived peptides, have been shown to combat fatigue by enhancing energy metabolism [53]. Specific polysaccharides that function through microbiota‐mediated mechanisms are gradually being uncovered: *Dendrobium*‐derived glucomannan improved insulin sensitivity by enriching *Parabacteroides distasonis* [54]; barley‐derived β‐glucan alleviated colonic inflammation via *Lactobacillus johnsonii* [55]; and inulin mitigated non‐alcoholic steatohepatitis by modulating gut metabolites [56]. Furthermore, the health benefits and mechanisms of natural extracts such as puerarin [57], ginsenoside Rg3 [58], and green tea polyphenols [59] are increasingly being clarified. With ongoing optimization of extraction techniques and better understanding of structure‐function relationships, natural extracts are poised to play an increasingly significant role in precision nutrition. Moreover, probiotics play a crucial role in maintaining gut microbial balance and promoting host health. Strains such as *Limosilactobacillus reuteri*, *Lactobacillus johnsonii*, and *Bifidobacterium pseudolongum* have been widely used to support digestive function, immune regulation, and metabolic homeostasis [55, 60, 61]. These beneficial microorganisms are typically isolated from fermented foods or human fecal samples, followed by extraction, purification, and rigorous validation of their safety and efficacy through clinical studies before scaled production [62]. Future studies are needed to expand the discovery of novel functional strains through culturomics and other high‐throughput approaches [63], supported by mechanistic and safety validation via in vitro and in vivo studies. With the increasing prevalence of technology and expansion of production scale, these ingredients may become more affordable and adaptable across different food products, supporting broader access to efficient and personalized nutrition strategies.

To address more complex or specific health demands, engineered foods and designed food ingredients may provide a higher degree of targeting and efficiency. The core of this approach is rational design based on structure‐activity relationships. For example, proteins or peptides can be designed with defined structural features to modulate specific bioactive pathways in the gut [64]. Similarly, ideal dietary fiber models facilitate precise control over structural parameters, such as monosaccharide composition and degree of polymerization, to achieve selective modulation of gut microbial ecosystems [65]. Delivery technologies, such as microencapsulation, can further improve the stability and intestinal release of probiotics by protecting them from gastric acid and other adverse conditions [66, 67]. However, these technologies are often associated with greater process complexity, higher production costs, and unresolved challenges in safety assessment and regulatory approval. Nevertheless, these solutions may offer valuable options for those who respond poorly to conventional interventions or have highly specific precision health needs.

### 3.2 Implementation Pathways

To better integrate precision nutrition into daily life, dietary recommendations should extend beyond nutrient composition and target selection to include practical and executable implementation strategies. A human‐centered precision nutrition framework should therefore provide diversified pathways, allowing people to choose solutions that align with their lifestyles, preferences, convenience needs, and health goals. One feasible implementation pathway is to provide personalized dietary meal plans, which offer considerable autonomy to those who enjoy cooking. With the intelligent upgrading of home kitchens, algorithm‐generated personalized recipes may be supported by smart refrigerators, precision electronic scales, and cooking robots, thereby reducing operational complexity and improving adherence [68]. For those with limited time or lower willingness to cook, health‐oriented meal delivery services may offer a more practical alternative. Central kitchens can prepare meals based on users’ precise nutritional needs and dietary preferences, combining whole foods and functional ingredients through standardized production processes. At the industrial level, flexible and intelligent food manufacturing may further support scalable personalization. Modern food industries are exploring smart manufacturing systems capable of adjusting ingredient composition and functional component ratios in response to different nutritional demands [69]. This approach may enrich the availability of products designed for specific nutrition needs while improving manufacturing precision and flexibility. For those requiring highly specialized nutritional support, emerging technologies such as 3D food printing provide additional possibilities [70, 71, 72]. Using precisely formulated food matrices or purified functional ingredients as raw materials, these technologies can generate foods with customized nutrient composition and texture through layer‐by‐layer fabrication.

Ultimately, precision nutrition should provide scientific recommendations regarding what to eat, while also offering multiple practical pathways for obtaining, preparing, and implementing these recommendations in real life. The long‐term value of human‐centered precision nutrition will lie in its capacity for continuous adaptation, using continuous feedback and iterative learning to generate recommendations that remain practical, relevant, and sustainable as health needs and lifestyles evolve.

## 4 Future Directions and Conclusion

**FIGURE 3:**
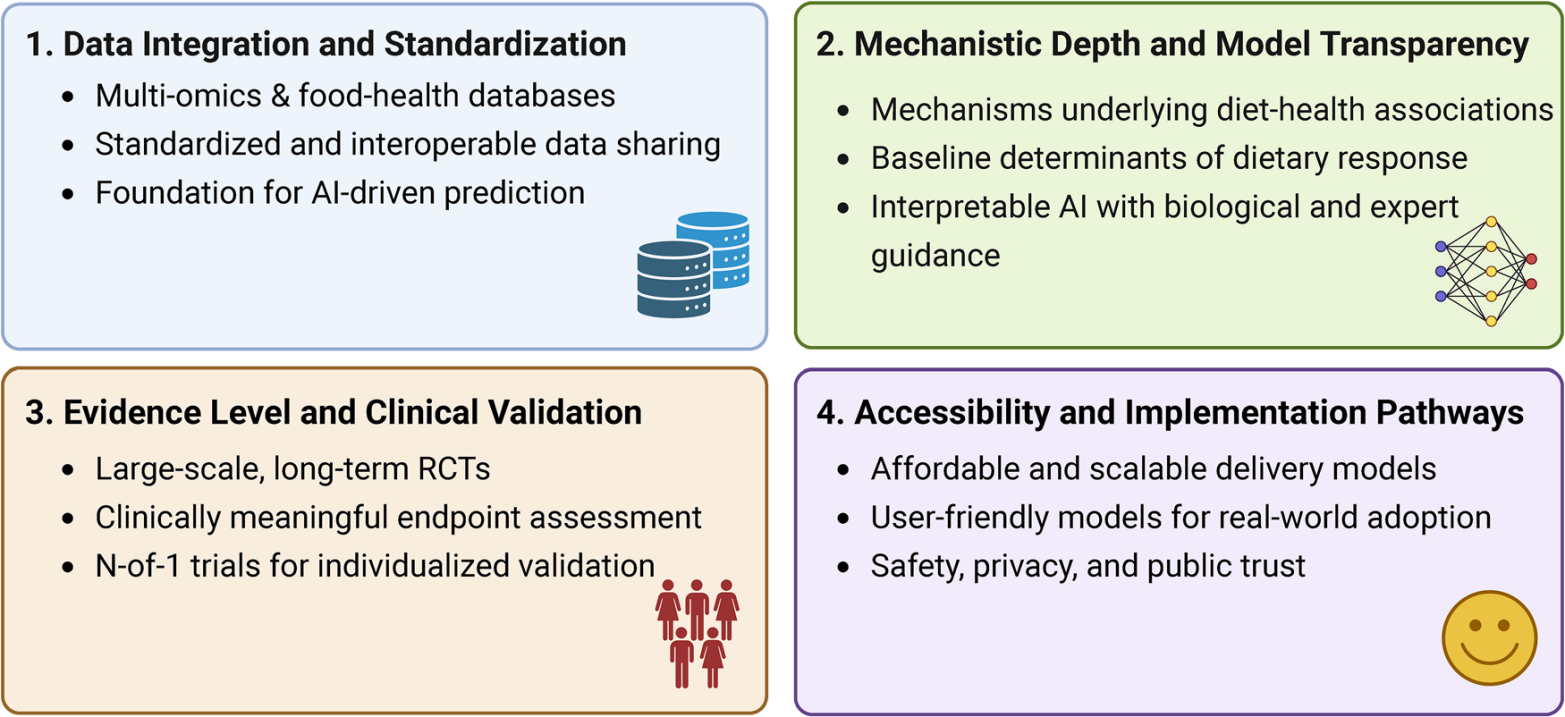
Future directions of precision nutrition. Future progress in precision nutrition will be advanced by four key priorities, including standardized data integration, mechanistic understanding and model transparency, rigorous clinical validation, and practical implementation pathways. Together, these directions can help translate AI‐driven precision nutrition from research to real‐world practice and support safe, scalable, and trusted adoption.

This article outlines a framework for human‐centered precision nutrition. By integrating AI for data analysis and a diverse toolbox of food‐based intervention strategies, the framework proposes a translational pathway from personalized data interpretation to practical dietary implementation. Despite its promising potential, translating this framework into reality faces several challenges, which also define critical future research priorities (Figure 3).

### 4.1 Data Integration and Standardization

A central bottleneck in current development is the heterogeneity of multi‐source and multi‐modal data in format and standards. This hinders the development and validation of powerful AI predictive models and limits the comparison and integration of findings across different studies. A major priority is therefore to establish large‐scale data alliances that span institutions, regions, and national borders. These efforts should integrate multi‐omics datasets that capture molecular‐level physiological states, databases describing the relationships between food components and health outcomes, and increasingly comprehensive evidence linking genetic variants to individual dietary responses. On this basis, unified data standards and sharing mechanisms should be developed to support interoperable research.

### 4.2 Mechanistic Depth and Model Transparency

Although numerous correlations between diet and health have been reported, the underlying causal molecular mechanisms remain insufficiently understood. A key challenge is the high variability in individual responses to dietary interventions, which is shaped by baseline characteristics such as specific single nucleotide polymorphisms (SNPs), gut microbiota composition, metabolic status, and other physiological traits. Future research should therefore construct a multi‐tiered evidence chain that links baseline features, dietary exposures, molecular responses, and health outcomes. This requires the use of in vitro culture systems and animal experiments to clarify how those specific characteristics influence intervention effects, followed by validation in large‐scale human studies to identify robust predictive biomarkers and causal pathways. The absence of such deep mechanistic insights directly limits the predictive accuracy and reliability of current AI models. Therefore, the development of interpretable AI tools should be prioritized, with expert knowledge incorporated into feature selection, model construction, causal interpretation, and recommendation design. This can improve the biological transparency and trustworthiness of dietary recommendations in real‐world applications.

### 4.3 Evidence Level and Clinical Validation

The current evidence base for precision nutrition is still developing, with important gaps in study scale, intervention duration, and clinically relevant endpoint assessment. Large‐scale, long‐term randomized controlled trials (RCTs) are therefore needed to evaluate whether precision nutrition strategies provide measurable benefits over conventional dietary approaches, particularly using clinically meaningful endpoints such as disease incidence, disease progression, metabolic improvement, or risk reduction in high‐risk populations. At the same time, future research should actively develop validation methods suitable for individualized interventions, such as N‐of‐1 trial designs. Conducting repeated crossover interventions within individuals can help verify the efficacy of specific dietary plans, thereby generating strong personalized evidence for highly tailored precision nutrition approaches.

### 4.4 Accessibility and Implementation

Achieving scalable deployment of precision nutrition requires future research to focus on its translational application pathways. This includes developing cost‐effective technical solutions, designing user‐friendly intervention models to facilitate adoption, and establishing appropriate regulatory frameworks to address emerging challenges such as food safety and data privacy. In parallel, effective science communication and public education will be essential for improving awareness and acceptance of precision nutrition.

In summary, precision nutrition represents a paradigm shift in nutritional science. We envision a future in which everyone can access truly tailored, culturally appropriate, and economically affordable dietary health solutions, which respect biological uniqueness and accommodate life realities, ultimately transforming precision nutrition from a cutting‐edge concept into a practical and scalable tool for improving population health.

## Author Contributions

**Shanshan Zhang**: Investigation, Writing – original draft. **Yonggan Sun**: Writing –review & editing. **Jiaqi Jiang**: Writing – review & editing. **Shaoping Nie**: Conceptualization, Funding acquisition, Supervision, Writing – review & editing.

## Conflicts of Interest

The authors declare no conflicts of interest.

## Data Availability

The authors have nothing to report.
